# Atractylodes lancea (Thunb.) DC. [Asteraceae] rhizome-derived exosome-like nanoparticles suppress lipopolysaccharide-induced inflammation in murine microglial cells

**DOI:** 10.3389/fphar.2024.1302055

**Published:** 2024-04-26

**Authors:** Kei Kawada, Tomoaki Ishida, Shumpei Morisawa, Kohei Jobu, Youichirou Higashi, Fuka Aizawa, Kenta Yagi, Yuki Izawa-Ishizawa, Takahiro Niimura, Shinji Abe, Mitsuhiro Goda, Mitsuhiko Miyamura, Keisuke Ishizawa

**Affiliations:** ^1^ Department of Clinical Pharmacology and Therapeutics, Tokushima University Graduate School of Biomedical Sciences, Tokushima, Japan; ^2^ Department of Clinical Pharmacy Practice Pedagogy, Tokushima University Graduate School of Biomedical Sciences, Tokushima, Japan; ^3^ Department of Pharmacy, Kochi Medical School Hospital, Kochi, Japan; ^4^ Department of Pharmacology, Kochi Medical School, Kochi University, Kochi, Japan; ^5^ Department of Pharmacy, Tokushima University Hospital, Tokushima, Japan; ^6^ Clinical Research Center for Developmental Therapeutics, Tokushima University Hospital, Tokushima, Japan; ^7^ Department of General Medicine, Taoka Hospital, Tokushima, Japan; ^8^ Center for Regional Sustainability and Innovation, Kochi University, Kochi, Japan

**Keywords:** Atractylodes lancea (Thunb.) DC. [Asteraceae] rhizome, microglia, exosome-like nanoparticle, Kampo medicine, microRNA, neuroinflammation

## Abstract

**Background:**

Exosome-like nanoparticles (ELNs) mediate interspecies intercellular communications and modulate gene expression.

**Hypothesis/Purpose:**

In this study, we isolated and purified ELNs from the dried rhizome of Atractylodes lancea (Thunb.) DC. [Asteraceae] (ALR-ELNs), a traditional natural medicine, and investigated their potential as neuroinflammatory therapeutic agents.

**Methods:**

ALR-ELN samples were isolated and purified using differential centrifugation, and their physical features and microRNA contents were analyzed through transmission electron microscopy and RNA sequencing, respectively. BV-2 microglial murine cells and primary mouse microglial cells were cultured *in vitro*, and their ability to uptake ALR-ELNs was explored using fluorescence microscopy. The capacity of ALR-ELNs to modulate the anti-inflammatory responses of these cells to lipopolysaccharide (LPS) exposure was assessed through mRNA and protein expression analyses.

**Results:**

Overall, BV-2 cells were found to internalize ALR-ELNs, which comprised three microRNAs (ath-miR166f, ath-miR162a-5p, and ath-miR162b-5p) that could have anti-inflammatory activity. Pretreatment of BV-2 cells with ALR-ELN prevented the pro-inflammatory effects of LPS stimulation by significantly reducing the levels of nitric oxide, interleukin-1β, interleukin-6, and tumor necrosis factor-α. Notably, the mRNA levels of *Il1b, Il6, iNos, ccl2*, and *cxcl10* in BV-2 cells, which increased upon LPS exposure, were significantly reduced following ALR-ELN treatment. Moreover, the mRNA levels of heme oxygenase 1, *Irf7, ccl12*, and *Irg1* also increased significantly following ALR-ELN treatment. In addition, pretreatment of primary mouse microglial cells with ALR-ELN prevented the pro-inflammatory effects of LPS stimulation by significantly reducing the levels of nitric oxide.

**Conclusion:**

Our findings indicate that ALR-ELNs exhibit anti-inflammatory effects on murine microglial cells. Further validation may prove ALR-ELNs as a promising neuroinflammatory therapeutic agent.

## 1 Introduction

Exosome-like nanoparticles (ELNs), which include plant-derived exosomes, mediate intercellular communications between different species (Woith et al., 2019; Kalluri and LeBleu, 2020). However, ELNs may have originally evolved in plants to support communication between plant cells and as a way of modulating innate immune defenses upon pathogen invasion (Ju et al., 2013). ELNs transport proteins, lipids, mRNAs, and microRNAs that are transferred into the host cells, where they act as extracellular messengers (Teng et al., 2018; Jia et al., 2021). Nanoparticles from edible plants (such as grape, grapefruit, ginger, and carrot) have anti-inflammatory properties and help maintain intestinal homeostasis (Mu et al., 2014); for example, ginger-derived nanoparticles protect against the development of liver-related diseases, including alcohol-induced damage (Zhuang et al., 2015). Additionally, ELNs have shown potential as valuable drug delivery tools owing to their biocompatibility, cellular uptake, and targeting capability (Dad et al., 2021).

Atractylodes lancea (Thunb.) DC. [Asteraceae] (*A. lancea*) is an important herb used in traditional natural medicine in East Asian countries, with its rhizome being used to treat rheumatic diseases, digestive disorders, night blindness, and influenza (Jun et al., 2018). The *A. lancea* rhizome exerts anticancer, anti-obesity, and anti-inflammatory effects (Koonrungsesomboon et al., 2014) owing to its sesquiterpene, sesquiterpenoid, polyethylene alkyne, and phytosterol contents (Jun et al., 2018).

Although natural metabolites modulate the response of microglial cells to pro-inflammatory agents (Nomura et al., 2017; Kawada et al., 2022), the specific role of ELNs in neuroinflammation remains largely unknown. BV-2 murine microglial cells treated with lipopolysaccharide (LPS) are widely used as an *in vitro* model for investigating the effects of natural metabolites on central nervous system disorders and inflammation (Kawada et al., 2022).

The purpose of this study was to evaluate the potential of *A. Lancea* rhizome-derived ELNs (ALR-ELNs) as neuroinflammatory therapeutic agents. First, we identified ALR-ELNs and characterized their role in the response of BV-2 and primary mouse microglial cells to LPS, including their impact on the regulation of genes involved in the inflammatory response and oxidative stress. Additionally, by combining publicly available data on microRNAs and ALR-ELN cargo, we aimed to identify candidate clinically valuable mRNA targets.

## 2 Materials and methods

### 2.1 Isolation and characterization of Atractylodes lancea-exosome-like nanoparticles

*A. lancea* rhizome samples were purchased from Tsumura (Tokyo, Japan) in November 2021, and authenticated using The Japanese Pharmacopoeia 18th edition. ALR-ELN samples were isolated and purified using differential centrifugation as previously described (Iitsuka et al., 2018). Briefly, *A. lancea* rhizome water-soluble substances were extracted by boiling 20 g of the herb sample in 400 mL of water for 30 min, followed by filtration. The extract was centrifuged at 8,000 × g for 5 min, and the supernatant was collected and centrifuged at 15,000 × g for 20 min. The supernatant was collected and filtered through a 0.8-µm filter (Millipore, Burlington, MA, United States). Then, the ALR-ELNs were extracted from the filtrate using an exoEasy Maxi Kit (Qiagen, Hilden, Germany) and stored at −70°C. A total of 10.4 ± 3.0 mg ELNs were isolated from 20 g of *A. lancea* rhizome crude samples. The chemical profile of ALR-ELNs according to the ConPhyMP statement (Heinrich et al., 2022) is provided in Supplementary Figure S1.

### 2.2 Transmission electron microscopy analysis

Nanoparticles were examined using a transmission electron microscope (JEM-2000EX; JEOL, Tokyo, Japan) operated at 100 kV at the Hanaichi UltraStructure Research Institute (Aichi, Japan). The diameter of the ELNs was measured and the size distribution was calculated.

### 2.3 RNA sequencing (RNA-seq) analysis

Total RNA was extracted from the ALR-ELNs using miRNeasy Mini Kit (Qiagen) according to the manufacturer’s protocol. RNA-seq analysis was performed by Macrogen (Tokyo, Japan). TruSeq Small RNA Library Prep Kit (Illumina, San Diego, CA, United States) was used for library preparation according to the manufacturer’s instructions. RNA samples were quality-tested using an Agilent 2100 Bioanalyzer (Agilent Technologies, Santa Clara, CA, United States). The libraries were sequenced using a HiSeq 2500 system (Illumina) and the resulting sequence reads were filtered to remove low-quality reads, repeated sequences, and adaptor sequences. Next, the reads were aligned to miRBase v22.1 (March 2022; http://www.mirbase.org) and RNAcentral v14.0 (March 2022; https://rnacentral.org) data to classify known microRNAs and other RNA molecules, and the identified reads were used for further analysis. The data are presented as the number of reads for each mature microRNA.

### 2.4 Confocal laser fluorescence microscopy

The ALR-ELN suspension was stained using the ExoSparkler Exosome Membrane Labeling Kit (Dojindo Laboratories, Kumamoto, Japan), according to the manufacturer’s protocol. Labeled ELNs (20 μg/mL) were added to BV-2 cells in a glass bottom dish and incubated for 3 h; 4′,6-diamidino-2-phenylindole was used as counterstain for the nuclei. The cells were observed under a confocal laser scanning microscope (FV-1000D/IX81; Olympus, Tokyo, Japan).

### 2.5 Cell viability

To evaluate the cytotoxic effects of ALR-ELNs, BV-2 cells (1 × 10^4^ cells/well) were cultured for 24 h and then treated with increasing concentrations of ALR-ELNs (5, 10, 20, and 40 μg/mL) for an additional 24 h. Next, the ALR-ELN-containing medium was removed, and 100 μL of medium containing 3-(4,5-dimethylthiazol-2-yl)-2,5-diphenyltetrazolium bromide (MTT) (0.5 mg/mL) was added and incubated for 1 h. Finally, the medium was removed, 100 μL of dimethyl sulfoxide was added to each well, and the absorbance was measured at 570 nm.

### 2.6 BV-2 microglial cell culture

Immortalized mouse microglial cells (BV-2; ABC-TC212S) were purchased from AcceGen Biotechnology (Fairfield, NJ, United States). The cells were maintained at 37°C and 5% CO_2_ in Dulbecco’s modified Eagle medium (DMEM) supplemented with 10% heat-inactivated endotoxin-free fetal bovine serum, 100 U/mL penicillin, and 0.1 mg/mL streptomycin. Exosome-depleted medium was obtained through ultracentrifugation at 110,000 × *g* overnight at 4°C and used in experiments involving ALR-ELNs.

BV-2 cells were grown in 24-well plates at a density of 5 × 10^4^ cells/well and incubated at 37°C. BV-2 cells were pretreated with ALR-ELNs (2.5, 5, 10, 20, and 40 μg/mL) or positive control (1.0 µM dexamethasone; Wako, Osaka, Japan) for 3 h and were then stimulated with 0.5 μg/mL LPS. For mRNA expression analysis, total RNA was extracted from BV-2 cells after 2 h of incubation. After 24 h of culture, the supernatant was collected and nitric oxide (NO) was measured using Griess assays, and interleukin (IL)-6, IL-1β, and tumor necrosis factor (TNF)-α levels were measured using commercial enzyme-linked immunosorbent assay kits (R&D Systems, Minneapolis, MN, United States) as per the manufacturer’s protocol.

### 2.7 Primary mouse microglial cell culture

Primary microglial cell cultures were prepared from mixed glial cultures from ICR mice (Japan SLC, Hamamatsu, Japan) as previously described (Higashi et al., 2011 Glia). All experimental protocols conformed to the guidelines of the National Institutes of Health (Guide for the Care and Use of Laboratory Animals, 1996) and were approved by the Committee for the Care and Use of Laboratory Animals at Kochi University (approval no. O-0009). In brief, cortices were dissected from 1-day-old mice. Cells were dissociated by mincing, followed by incubation in papain and DNase for 10 min at 37°C. After centrifugation for 5 min at 500 × *g*, the cells were resuspended and triturated with a pipette into DMEM supplemented with 10% fetal bovine serum (FBS) (Biowest, Miami, FL) and 2 mM glutamine. Cells were plated on 6-well plates at a density of 6.4 ×10^5^ cells/well and maintained in a CO_2_ incubator. The medium was changed after 3 days *in vitro* and once per week thereafter. This procedure resulted in cultures consisting of astrocytes and microglia cells. After 2 weeks *in vitro*, microglia were harvested by mildly shaking the cultures and collecting the floating cells. The cells were replated at a density of 5 × 10^5^ cells/well on 24-well plates to obtain pure microglial cultures. The microglial cultures were used for experiments 2 days after replating (*in vitro* day 16). Each culture well was visually inspected by phase contrast microscopy before use, and wells containing >30% activated microglia were not used in the experiments.

Primary microglial cells were pretreated with ALR-ELNs (20 μg/mL) or positive control (1.0 µM dexamethasone) for 3 h and then stimulated with 0.5 μg/mL LPS. After 24 h of culture, the supernatant was collected and NO was measured using Griess assays.

### 2.8 mRNA expression analysis by real-time quantitative polymerase chain reaction (RT-qPCR)

We used real-time PCR to identify several genetic changes related to inflammatory pathways. This was based on a study that employed dual RNA sequencing to analyze how gene expression differs in BV-2 microglial cells when exposed to inflammatory stimuli (Das et al., 2015). Total RNA was extracted from BV-2 cells using the RNeasy Mini Kit (Qiagen), and then reverse transcription was conducted using the PrimeScript RT Reagent Kit (Takara Bio, Kusatsu, Japan). The conversion from RNA to cDNA was performed with the PrimeScript RT Reagent Kit and PCR Thermal Cycler Dice (Takara Bio). Each cDNA sample was mixed with forward and reverse primers and the THUNDERBIRD SYBR qPCR mix (Toyobo, Osaka, Japan) as per the manufacturer’s instructions. The PCR mixture contained 1 µL cDNA, 5 µL THUNDERBIRD SYBR qPCR mix, 0.2 µL PCR primers, and 3.6 µL RNase-free water. PCR was conducted using an Applied Biosystems StepOnePlus system as previously described (Yoshioka et al., 2023). This involved 45 cycles of denaturation at 95°C for 15 s, followed by annealing and extension at 60°C for 1 min. Initial analysis was performed using StepOnePlus version 2.3 (Applied Biosystems, Foster City, CA, United States). The relative fold change in gene expression, compared to the control group, was determined using mouse GAPDH as an internal reference. The primer set used for PCR is provided in Supplementary Table S1.

### 2.9 Protein analysis

BV-2 cells were seeded in 24-well plates at a density of 1.0 × 10^5^ cells/well and incubated for 24 h. Afterward, the cells were pretreated with ALR-ELNs (20 μg/mL) for 3 h and stimulated with 0.5 μg/mL LPS, after which the cells were incubated for 12 h. Then, the cells were collected and washed with phosphate-buffered saline followed by the addition of RIPA buffer (Santa Cruz Biotechnology, Dallas TX, United States). Protein concentrations were determined by BCA protein assay (Thermo Fisher Scientific).

Automated western blots were performed using the Wes Western Blot System (ProteinSimple, San Jose, CA, United States) according to the manufacturer’s protocol and recommendations. Cell lysates were diluted to 0.2 mg/mL, and a size assay was run on a 25-well plate. The assay parameters were as follows: 25 min separation time, 375 V, 5 min antibody diluent, 30 min primary antibody incubation, and 30 min secondary antibody incubation. The following primary antibodies were used: anti-iNOS (dilution 1:25; mouse, R&D Systems) and anti-β-actin (1:250; rabbit, Cell Signaling Technology, Danvers, MA, United States). Densitometric analysis was performed using the Compass software (ProteinSimple) and protein quantification was conducted using the area under the curve calculation method.

### 2.10 Statistical analysis

All statistical analyses were performed using EZR version 1.29 (Saitama Medical Center, Jichi Medical University, Saitama, Japan) (Nomura et al., 2017). Data are expressed as mean ± standard deviation (SD). One-way analysis of variance (ANOVA) was performed to examine the significance of the differences between treatments. Subsequently, multiple comparison tests were performed using Tukey’s test. Statistical significance was set at *p* < 0.05.

## 3 Results

### 3.1 Characterization of ELNs from A. lancea rhizome

**FIGURE 1:**
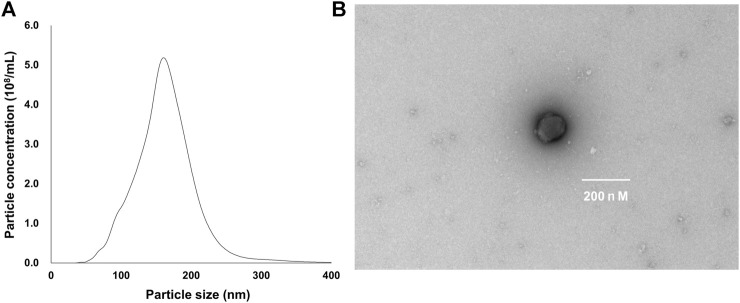
Characterization of *Atractylodes lancea* rhizome-derived exosome-like nanoparticles (ALR-ELNs). **(A)** Size and yield, and **(B)** morphology of the nanoparticles were determined using transmission electron microscopy.

TEM analysis showed that the ALR-ELNs were 50–365 nm in size and had a round shape (Figures 1A,B). To investigate the expression profile of miRNAs in A-ELNs, total RNA of A-ELNs was extracted. Small RNA libraries were then constructed and sequenced to generate a total of 6,848,533 raw reads. After applying a series of stringent filters, the remaining 269,832 reads (3.94% of raw reads) from all libraries were considered reliable miRNA candidates. Additionally, a comparison of the miRNA sequences found in A-ELNs with the mature miRNA library of *Arabidopsis thaliana* revealed four known miRNAs (ath-miR166f, ath-miR162a-5p, ath-miR162b-5p, and ath-miR396b-5p). Information on the mature miRNA sequences of the three detected miRNAs is shown in Table 1.

**TABLE 1:** MicroRNAs identified in *Atractylodes lancea* rhizome-derived exosome-like nanoparticles.

MicroRNA	Mature miRNA sequence	Length (nt)	Number of reads
ath-miR166f	ucg​gac​cag​gcu​uca​uuc​c	19	46
ath-miR162a-5p	ucg​aua​aac​cuc​ugc​auc​ca	20	2
ath-miR162b-5p	ucg​aua​aac​cuc​ugc​auc​ca	20	2

### 3.2 Effects of ALR-ELNs on BV-2 cell viability

**FIGURE 2:**
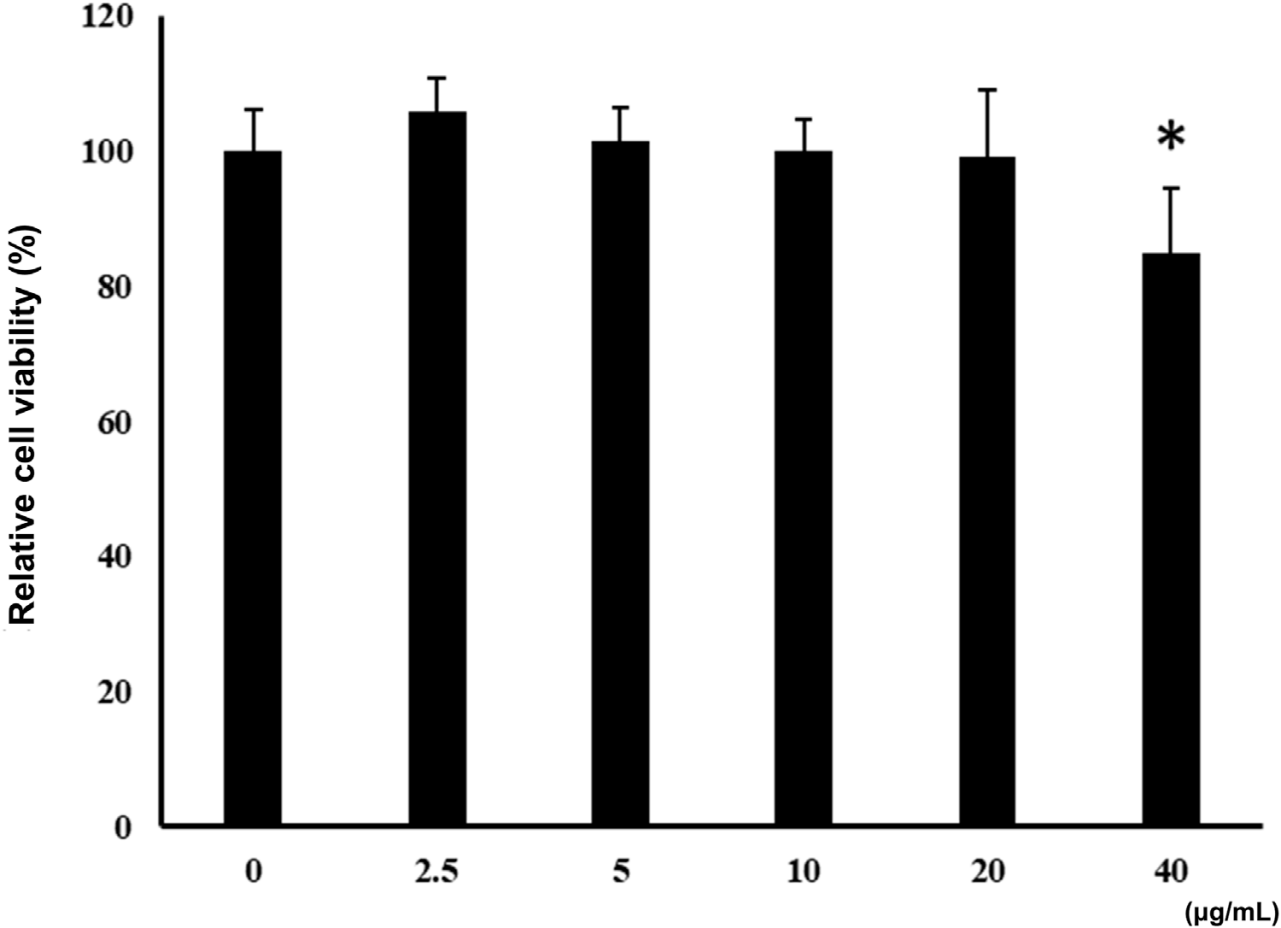
Uptake of ALR-ELNs by murine microglial cells. BV-2 cells were incubated with labeled ELNs (green) for 3 h and observed using confocal microscopy. 4′,6-Diamidino-2-phenylindole (red) was used as nuclei counterstain.

**FIGURE 3:**
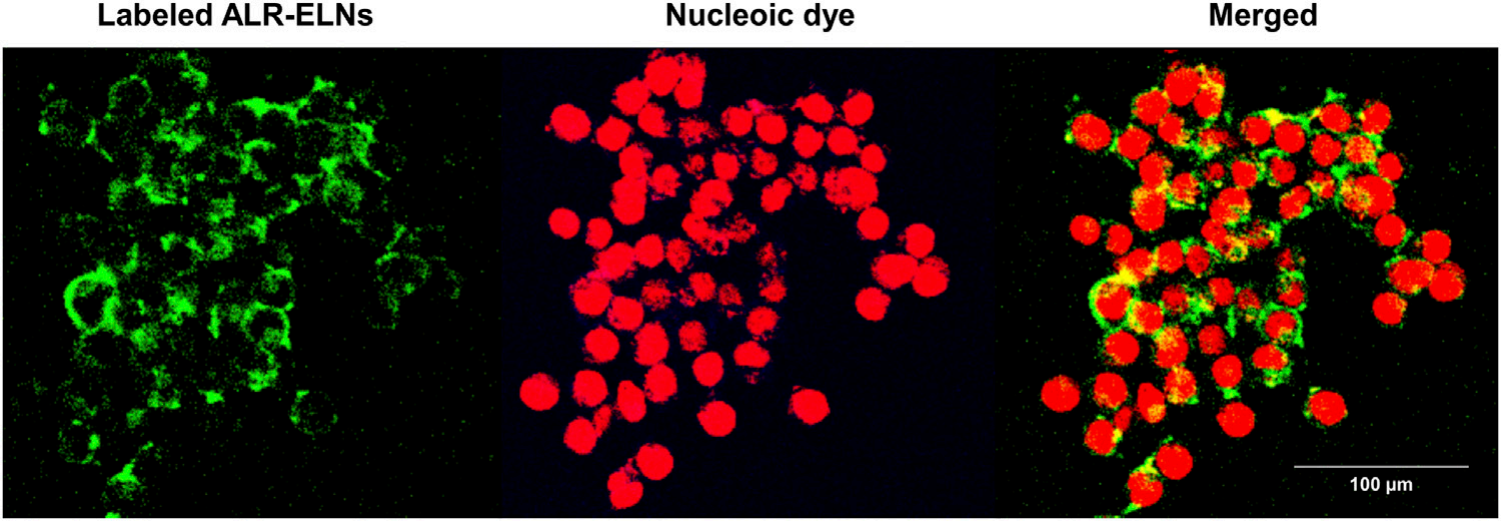
Effects of ALR-ELNs on the viability of murine microglial cells. BV-2-cells (1 × 10^4^ cells/well) were incubated for 24 h with increasing concentrations of ELNs (2.5, 5, 10, 20, and 40 μg/mL). Cell viability was determined using the MTT assay. Values are expressed as mean ± SD. *p < 0.05 by one-way ANOVA followed by Tukey’s test.

Incubation of BV-2 cells for 3 h with the isolated ALR-ELNs showed that the nanoparticles were taken up by the cells (Figure 2), which confirmed that the nanoparticles could directly interact with the cells. Moreover, the ELNs did not exert significant toxicity on BV-2 cells when administered at 5–20 μg/mL, whereas significant toxicity was observed at 40 μg/mL as compared with control phosphate-buffered saline-treated cells (84.9% ± 9.7%; *p* = 0.012) (Figure 3).

### 3.3 Effects of ALR-ELNs on LPS-induced release of inflammatory mediators by BV-2 cells

**FIGURE 4:**
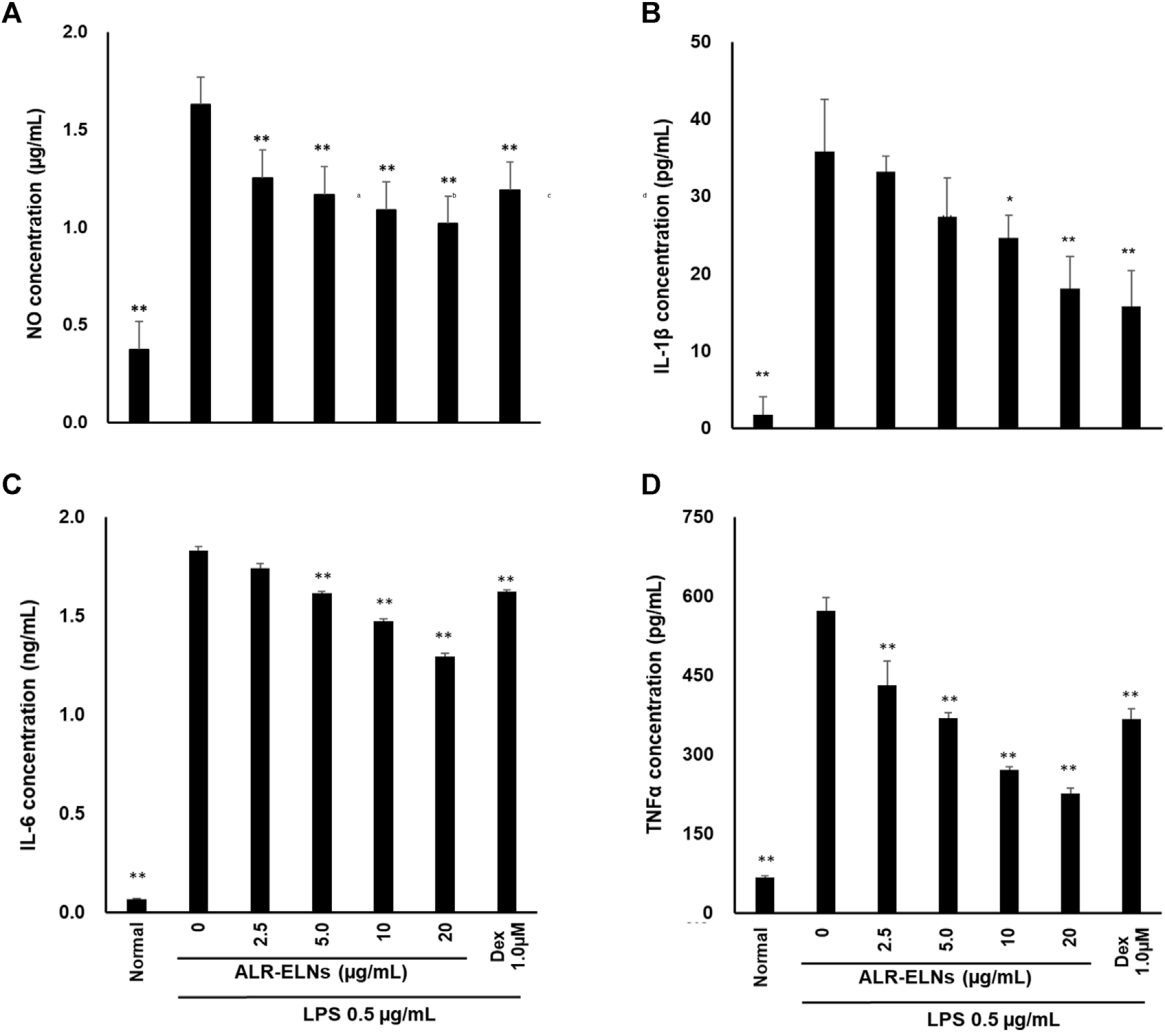
Effects of ALR-ELNs on lipopolysaccharide (LPS)-induced release of inflammatory mediators by BV-2 cells. The cells were pretreated with ALR-ELNs (0–20 μg/mL) or dexamethasone (1 μM) for 3 h and stimulated with 0.5 μg/mL LPS for 24 h. The levels of the indicated cytokines were determined by enzyme-linked immunosorbent assays. **(A)** Nitric oxide (NO): F 6,21 = 85.0, *p* < 0.01; **(B)** Interleukin (IL)-1β: F 6,21 = 27.0, *p* < 0.01; **(C)** IL-6: F 6,21 = 2941, *p* < 0.01; and **(D)** tumor necrosis factor (TNF)-α: F 6,21 = 11511, *p* < 0.01. Values are expressed as mean ± SD. **p < 0.01 vs. LPS treatment alone by one-way ANOVA followed by Tukey’s test.

**FIGURE 5:**
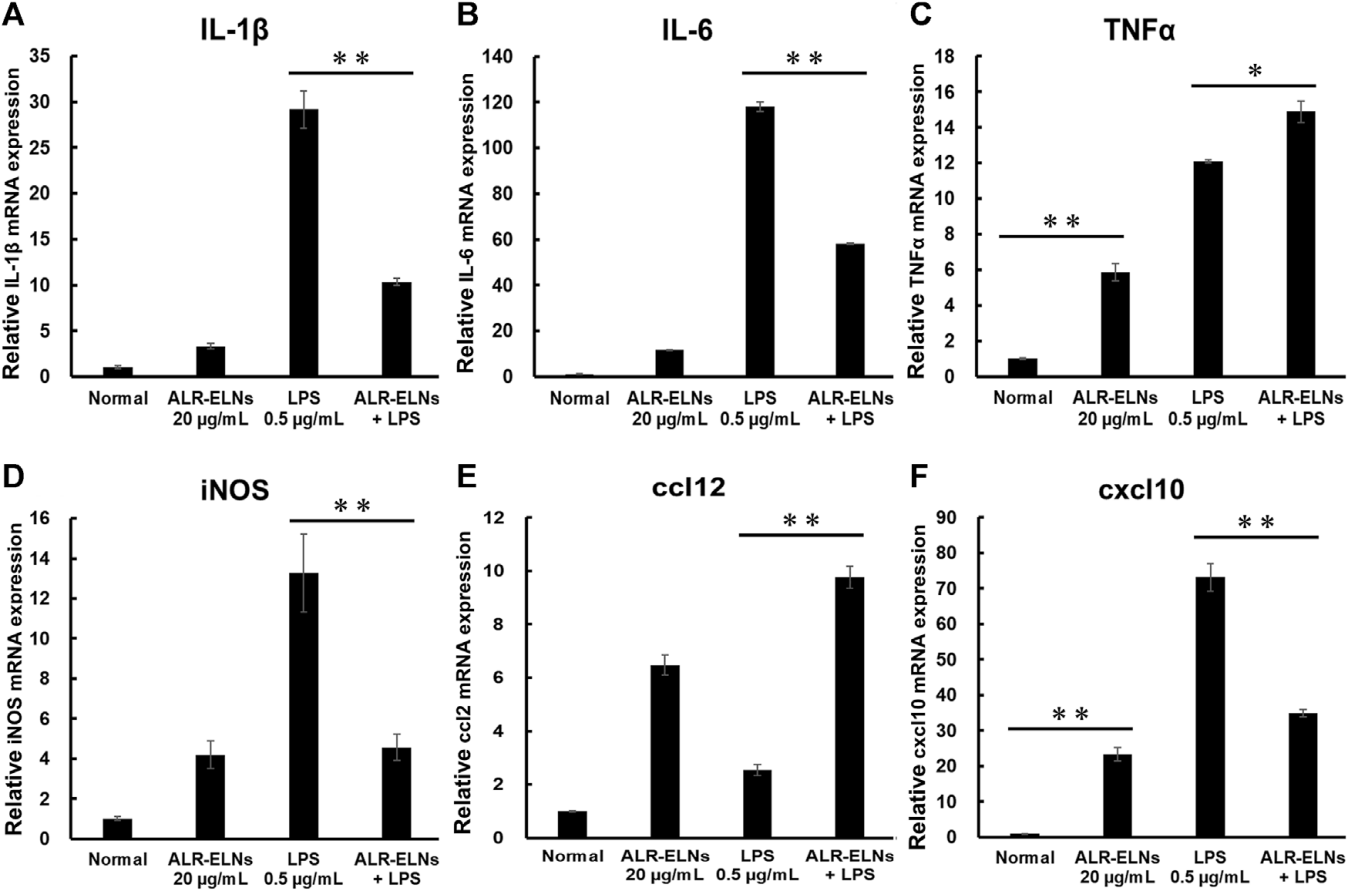
Effects of ALR-ELNs on LPS-induced mRNA levels of proinflammatory mediators in BV-2 cells. The cells were pretreated with 20 μg/mL ALR-ELNs for 3 h and stimulated with 0.5 μg/mL LPS for 2 h **(A)** *Il1b*: F 3,12 = 97.8, *p* < 0.01; **(B)** *Il6*, F 3,12 = 111.2, *p* < 0.01; **(C)** *Tnfα*, F 3,12 = 169.2, *p* < 0.01; **(D)** *iNos*: F 3,12 = 15.7, *p* < 0.01; **(E)** *ccl2*: F 3,12 = 59.6; *p* < 0.01 and **(** **F** **)** *cxxl10*: F 3,12 = 122.8, *p* < 0.01. Values are expressed as mean ± SD. **p < 0.01 vs. LPS treatment alone by one-way ANOVA followed by Tukey’s test.

**FIGURE 6:**
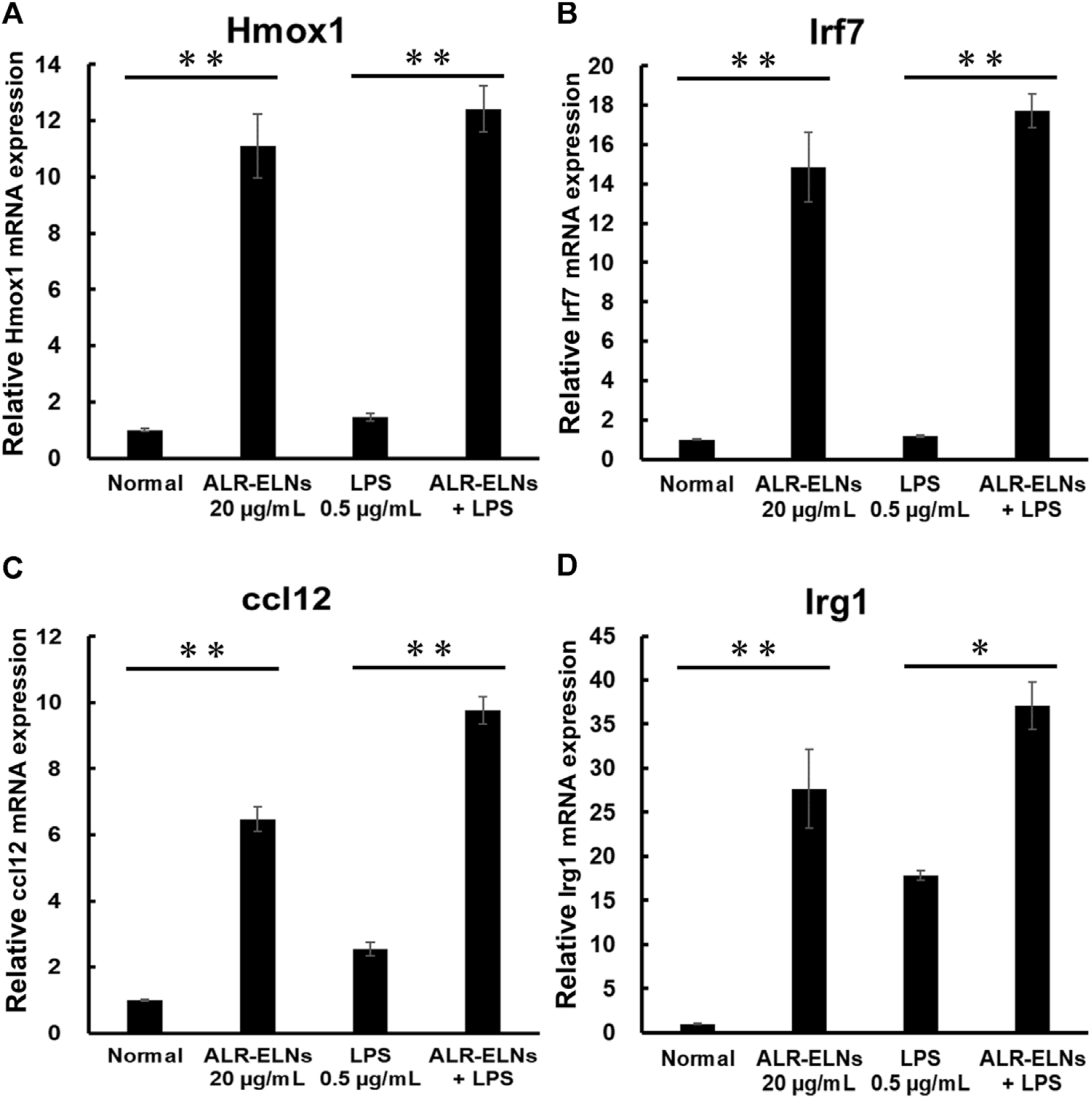
Effects of ALR-ELNs on LPS-induced mRNA levels of anti-inflammatory mediators in BV-2 cells. The cells were pretreated with 20 μg/mL ALR-ELNs for 3 h and stimulated with 0.5 μg/mL LPS for 2 h **(A)** *Hmox1*: F 3,12 = 50.2, *p* < 0.01; **(B)** *Irf7*, F 3,12 = 54.7, *p* < 0.01; **(C)** *ccl12*: F 3,12 = 113.2, *p* < 0.01; and **(D)** Irg1 F 3,12 = 23.2; *p* < 0.01. Values are expressed as mean ± SD. **p < 0.01 vs. LPS treatment alone by one-way ANOVA followed by Tukey’s test.

**FIGURE 7:**
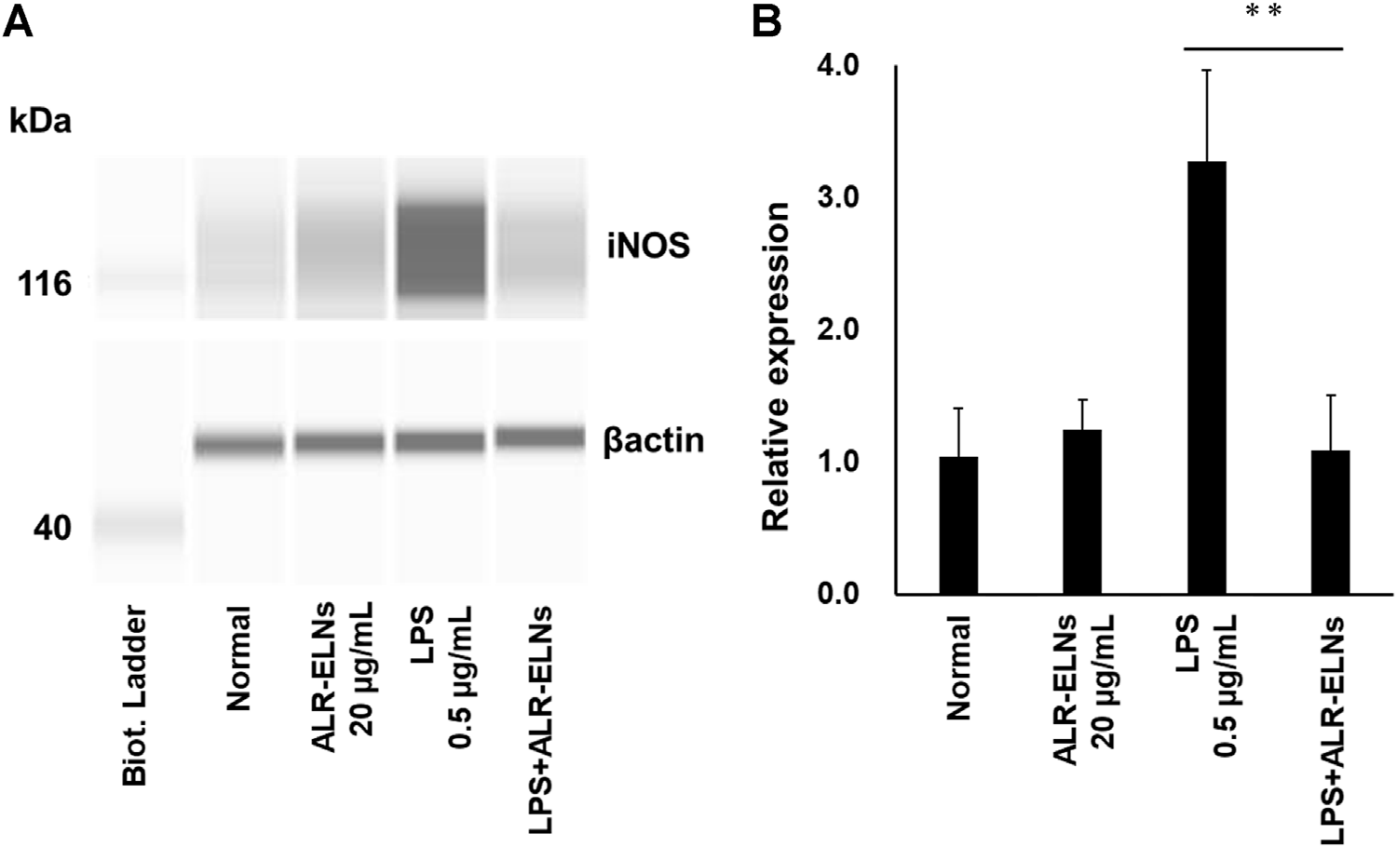
Effects of ALR-ELNs on LPS-induced levels of iNOS in BV-2 cells. The cells were pretreated with 20 μg/mL ALR-ELNs for 3 h and stimulated with 0.5 μg/mL LPS for 12 h. **(A)** Western blotting analysis of iNOS levels. Expression of β-actin served as loading control. **(B)** Densitometric analysis of **(A)**. Values are expressed as mean ± SD. F 3,12 = 22.5, **p < 0.01 vs. LPS treatment alone by one-way ANOVA followed by Tukey’s test.

We investigated the anti-inflammatory effects of ALR-ELN on BV-2 cells. Pretreatment of BV-2 cells with 5–20 μg/mL ALR-ELNs for 3 h significantly prevented the pro-inflammatory effects of 0.5 μg/mL LPS stimulation, as indicated by the significantly reduced levels of secreted NO, IL-1β, IL-6, and TNF-α (Figure 4). Further analysis of BV-2 cells using RT-qPCR confirmed that the expression of *Il1b*, *Il6*, *iNos*, *ccl2*, and *cxcl10* increased after LPS treatment (0.5 μg/mL for 2 h), an effect that was significantly counteracted by ALR-ELN pretreatment (20 μg/mL for 3 h) (Figures 5A,B; 5D–F). However, *Tnfα* levels were increased after both ALR-ELN pretreatment and LPS stimulation (Figure 5C). Additionally, mRNA expression of *Hmox1*, *Irf7*, *ccl12*, and *Irg1* in BV-2 cells significantly increased following ALR-ELN treatment (Figures 6A–D).These results were also verified through Western blot analysis of BV-2 cells pretreated with 20 μg/mL ALR-ELNs for 3 h and stimulated with 0.5 μg/mL LPS for 12 h. Inducible nitric oxide synthase (iNOS) expression was increased upon LPS treatment, an effect that was significantly counteracted by ALR-ELN treatment (*p* < 0.01) (Figure 7), whereas ALR-ELNs alone had no significant effect on control (non-LPS-stimulated) BV-2 cells.

### 3.4 Effects of ALR-ELNs on LPS-induced release of inflammatory mediators by primary microglial cells

**FIGURE 8:**
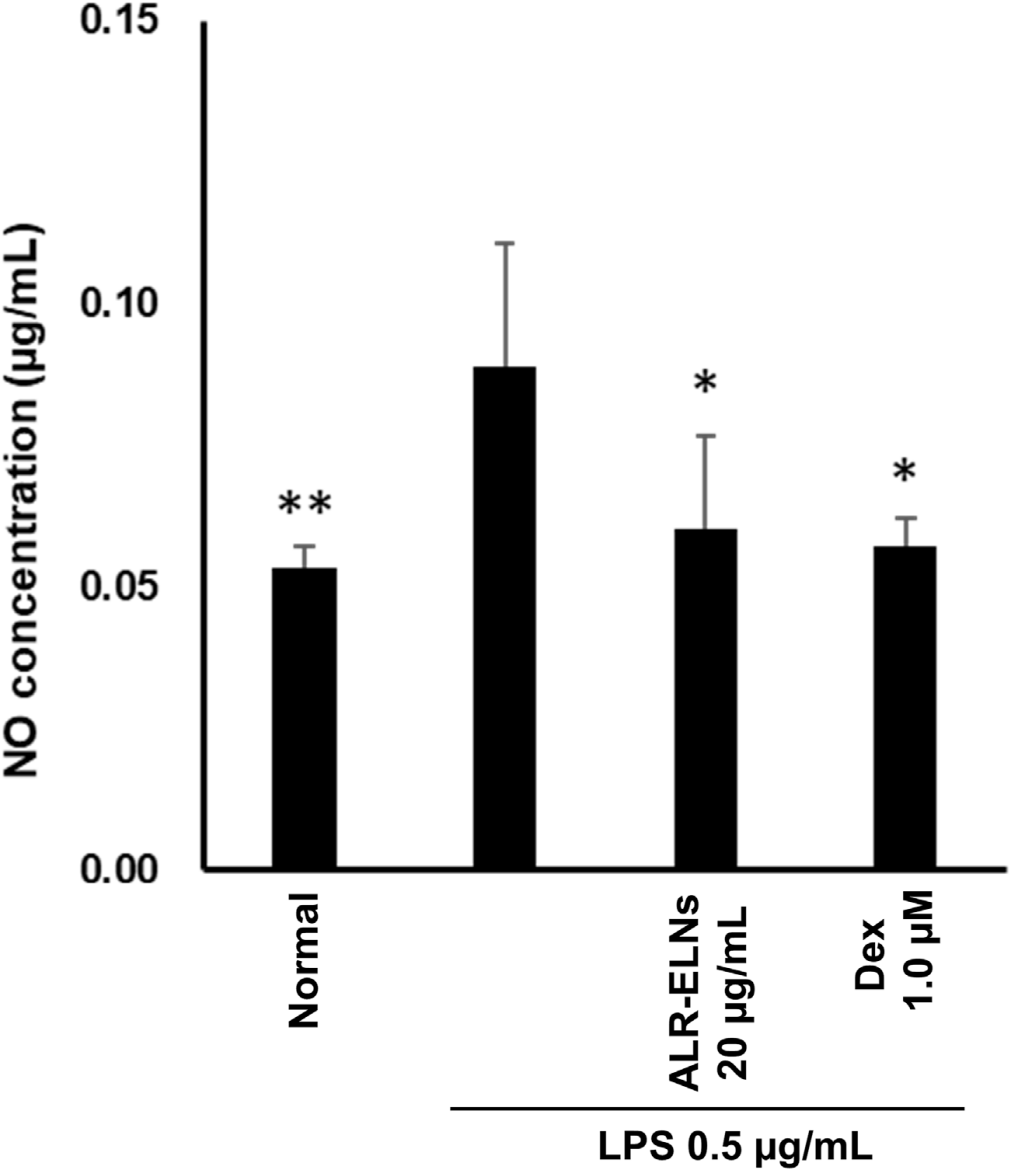
Effects of ALR-ELNs on LPS-induced levels of nitric oxide (NO) in primary microglial cells. The cells were pretreated with ALR-ELNs (20 μg/mL) or dexamethasone (1 μM) for 3 h and stimulated with 0.5 μg/mL LPS for 24 h. NO: F 5,18 = 4.59, *p* < 0.01.

We investigated the anti-inflammatory effects of ALR-ELN on primary cultured mouse microglia. Pretreatment of primary microglial cells with 20 μg/mL ALR-ELNs and that of the positive control with 1.0 µM dexamethasone for 3 h significantly prevented the pro-inflammatory effects of 0.5 μg/mL LPS stimulation, as indicated by the significantly reduced levels of secreted NO (Figure 8).

## 4 Discussion

*A. lancea* rhizome, which is widely distributed in China, is a natural medicine derived from *Atractylodes lancea* and *Atractylodes chinensis* from the aster family (*Compositae*) that is used as an ingredient in many types of Kampo medicines. Natural medicines, such as *A. lancea* rhizome, possess neuropharmacological efficacy commonly attributed to the action of its constituent secondary metabolites (Ikarashi and Mizoguchi, 2016; Kawada et al., 2022). The general pharmacological effects of natural medicines are thought to result from secondary metabolites, although it may not be sufficient to explain the strength of the effects. Because plant-derived extracts contain ELNs that may exhibit pharmacological activity (Woith et al., 2019; Kalluri and LeBleu, 2020), they may be responsible for some of the pharmacological effects of Kampo medicines. Herein, we investigated the presence of ELNs in *A. lancea* rhizome. To the best of our knowledge, this is the first study characterizing the uptake of ALR-ELNs by mammalian cells and their ability to modify the gene expression profile of microglial cells.

ELNs involved in plant cell–cell communication may potentially regulate the innate immune system of plants and transport bioactive molecules, including mRNAs, microRNAs, and proteins, to recipient cells in different contexts (Teng et al., 2018). However, few studies have investigated the therapeutic potential of ELNs (Iitsuka et al., 2018). In this study, we isolated, purified, and characterized ELNs from dried *A. lancea* rhizome, adding to the previous knowledge on ELN extraction from raw fruits and vegetables (Ju et al., 2013; Zhuang et al., 2015; Pérez-Bermúdez et al., 2017; De Robertis et al., 2020). Although ELN isolation from dried herbs can be challenging, possibly because the nanoparticles become unstable during the drying process because of their lipid bilayer and membrane proteins (Suharta et al., 2021), we demonstrated that active ELNs can be extracted from dried natural medicinal herbs. Therefore, ELNs are resistant to drying and may contribute to the efficacy of dried natural medicines.

In this study, we successfully isolated ALR-ELNs ranging from 50 to 365 nm in size (Figures 1A,B). The size of these ALR-ELNs resembled that of previously described plant-derived ELNs (Kim et al., 2022). Previous studies have revealed similarities between grape-derived ELNs and mammalian exosomes, such as shared proteins (such as heat shock proteins and aquaporins) and lipids rich in phosphatidic acids and phosphatidylethanolamines (Dad et al., 2021). They also share comparable nano-sizes and vesicle structures. These common characteristics enable grape ELNs to traverse the gut, stimulate stem cell growth, and contribute to the regeneration of intestinal epithelial tissue (Ju et al., 2013). Additionally, ELNs have distinct pharmacokinetics, and their lipid bilayer structure and composition affect their entry into cells (Yepes-Molina et al., 2020). The present study demonstrated that ALR-ELNs directly interacted with and were taken up by BV-2 cells from mouse microglia (Figure 2). Furthermore, the presence of mature miRNAs in ALR-ELNs is consistent with findings from previous studies (De Robertis et al., 2020; Kim et al., 2022), suggesting that mature miRNAs possess pharmacological activity. Although earlier reports did not focus on microglial cells, our study suggests that ELNs could be beneficial in new areas.

Inflammation promoted by microglia is associated with the development of various neurological diseases, including multiple sclerosis (MS), Alzheimer’s disease, Parkinson’s disease, and depression (Minter et al., 2016; Bright et al., 2019; Troubat et al., 2021). Indeed, in such conditions, microglia release inflammatory factors (such as IL-1β, IL-6, and NO) that damage the nerve cells (Smith et al., 2012). NO is a free radical synthesized by iNOS that is involved in microglia-mediated inflammatory processes in the central nervous system (Subedi et al., 2021). We evaluated the effects of ALR-ELNs on microglial inflammation using LPS-stimulated BV-2 cells as an *in vitro* model. ALR-ELN pretreatment significantly reduced the mRNA levels of inflammatory cytokines (IL-1β, IL-6, and iNOS). ALR-ELN pretreatment caused a decrease in TNFα mRNA levels in the 24-h cultured supernatant after LPS stimulation (Figure 4D). However, in cells collected 2 h after LPS stimulation, ALR-ELN pretreatment led to an increase in TNFα mRNA levels (Figure 5C). This could be attributed to TNFα′s rapid response to stimulation, resulting in a transient increase with both LPS and ALR-ELN pretreatment (Das et al., 2015). However, ALR-ELN pretreatment may inhibit TNFα expression following LPS stimulation due to its anti-inflammatory effect. ALR-ELN pretreatment resulted in a significant reduction in the mRNA levels of the chemokines ccl2 and cxcl10. Chemokines, recognized as inflammatory cytokines, play a crucial role in regulating inflammation. Elevated levels of these molecules are linked to disease progression and severe inflammatory conditions, including MS (Murdoch and Finn, 2000). Ccl2 is particularly important in neuroinflammatory diseases and is a potential target for treatment (Conductier et al., 2010). It is highly expressed in microglia, astrocytes, and other inflammatory cells during MS (Banisor et al., 2005). Cxcl10, another chemokine, is associated with infectious and inflammatory diseases, contributing to T cell-mediated inflammation in the central nervous system. Moreover, Cxcl10 plays a role in inflammatory demyelinating diseases such as MS by promoting leukocyte trafficking in the brain, leading to the destruction of myelin sheaths or neurons (Shen et al., 2006). The levels of NO and pro-inflammatory cytokines are closely related to heme oxygenase (HO)-1 expression (Campbell et al., 2021). HO-1 suppresses LPS-induced inflammation in BV-2 cells (Lee et al., 2017). In agreement with these findings, ALR-ELN treatment significantly induced the expression of *Hmox1* regardless of the presence of LPS. Hence, it is reasonable to speculate that HO-1 mediates the anti-inflammatory effects of ALR-ELNs. In addition, HO-1 contributes to other mechanisms that suppress the inflammatory response triggered by LPS, such as the SHP2–NLRP3 and Nrf2–ARE pathways (Buendia et al., 2016; Guo et al., 2017). Furthermore, ALR-ELN treatment increased the mRNA levels of *Irf7*, *ccl12*, and *Irg1*. In microglia, Irf7 promotes both anti- and pro-inflammatory phenotypes and activation of Irf7 through IFN-β restores the anti-inflammatory phenotype (Cohen et al., 2014). Ccl12 reduces neuroinflammation and inhibits inflammasome activation in LPS-stimulated BV2 cells (Roosen et al., 2021). Irg1 regulates the immune metabolism in inflammation and infectious diseases (Wu et al., 2020). Consequently, the anticipated anti-inflammatory impact of ALR-ELNs is likely to involve various signals, and additional research is necessary to gain a deeper understanding of the mechanisms behind the anti-inflammatory effects of ALR-ELNs.

Active oxygen may be involved in the anti-inflammatory effects of ALR-ELNs in BV-2 cells, similar to blueberry-derived ELNs that contain miR162 and exhibit antioxidant activity (De Robertis et al., 2020). Thus, these microRNAs may mediate the inhibitory effects of ALR-ELNs on LPS-induced inflammation in microglial cells. Future studies should focus on the use of ELNs as vectors for active metabolites, such as drugs, RNAs, proteins, and lipids. In addition, ALR-ELNs were found to contain multiple microRNAs including miR166, which is predicted to target the pro-inflammatory molecules B-cell lymphoma 2, VAV1, and IL2 receptor alpha with high affinity (Minutolo et al., 2020). In particular, VAV1 upregulates the expression of inflammatory mediators (Miletic et al., 2007) and may play a role in the anti-inflammatory effects of ALR-ELNs. However, VAV1 does not affect iNOS or IL-6 (Miletic et al., 2007); therefore, ALR-ELNs may have an alternative mode of action independent of VAV1, which warrants further investigations. In addition, ALR-ELNs did not contain beta-eudesmol, hinesol, atractylone, and atractylodin, which are the major constituents of *A. lancea* rhizome (Koonrungsesomboon N et al., 2014), but 7-methoxycoumarin was found to be a component of ALR-ELNs (Supplementary Figure S1). Methoxycoumarin is found in various plants and has anti-inflammatory effects on microglia (Togna et al., 2014; Kirsch et al., 2016; Kang and Hyun, 2020). However, ALR-ELN may contain components other than methoxycoumarin, and future studies should investigate whether the components of ALR-ELNs contribute to the anti-inflammatory effects.

This study has several limitations. First, some quality indicator data for the ELNs, such as the PDI, were not available. These data are considered necessary for future animal studies and clinical applications (Danaei et al., 2018), as these act as indicators that affect the *in vitro* and *in vivo* behavior of the nanoparticles. Second, we performed a comprehensive analysis of microRNAs through RNA sequencing and found that several characteristic microRNAs are transported by the ALR-ELNs; however, these microRNAs have not been quantified. Therefore, it is necessary to investigate them in the future using RT-qPCR. Third, despite the collective evidence that ALR-ELNs can be taken up by BV-2 cells, this alone does not prove complete cellular uptake. In the future, it will be necessary to confirm that the ALR-ELNs are incorporated into cells using more reliable methods.

## 5 Conclusion

In summary, our study reports for the first time the anti-inflammatory effects of ALR-ELNs on LPS-stimulated microglia. We believe that the findings of this study provide additional insights into the pharmacological efficacy of natural medicines via their ELNs and valuable bioactive agents. Future studies of ALR-ELN action *in vivo* may prove ALR-ELNs to be promising neuroinflammatory therapeutic agents.

## Data Availability

The raw data supporting the conclusion of this article will be made available by the authors, without undue reservation.
