# Supplementary material for: Atractylodes lancea (Thunb.) DC. [Asteraceae] rhizome-derived exosome-like nanoparticles suppress lipopolysaccharide-induced inflammation in murine microglial cells
Source: Front Pharmacol. 2024 Apr 26;15:1302055. doi: 10.3389/fphar.2024.1302055 (PMC11082290; doi:10.3389/fphar.2024.1302055)
Supplement: Supplementary file 1 [file Table1.DOCX]

**Supplemental Table S1. Primer sequences.**

| Mouse *Cxcl10* | Forward | 5’ -CATCCTGCTGGGTCTGAGTGG -3’ |
| --- | --- | --- |
|  | Reverse | 5’ -CCCTATGGCCCTCATTCTCAC -3’ |
| Mouse *Ccl2* | Forward | 5’ -GGAGAGCTACAAGAGGATCACCAG -3’ |
|  | Reverse | 5’ -GTCTGGACCCATTCCTTCTTGG -3’ |
| Mouse *Il-1β* | Forward | 5’ -GTGAAATGCCACCTTTTGACAGTGATG -3’ |
|  | Reverse | 5’ -CTTGTTGATGTGCTGC -3’ |
| Mouse *Il-6* | Forward | 5’ -GTCCTTCCTACCCCAATTTCCAATGC -3’ |
|  | Reverse | 5’ -GGTCTTGGTCCTTAGCCACTCCTTC -3’ |
| Mouse *Tnf-α* | Forward | 5’ -GACCCTCACACTCAGATCATCTTCTC -3’ |
|  | Reverse | 5’ -GCTACGACGTGGGCTACAGG -3’ |
| Mouse *iNOS* | Forward | 5’ -GCTACTGAGACAGGGAAGTCTGAAG -3’ |
|  | Reverse | 5’ -GTCACCACCAGCAGTAGTTGCTC -3’ |
| Mouse *Ccl12* | Forward | 5’ -CAGGAGAATCACAAGCAGCCAGTG -3’ |
|  | Reverse | 5’ -GAGACGTCTTATCCAAGTGGTTTATGG -3’ |
| Mouse *Irf 7* | Forward | 5’ –CCCTCTGCTTTCTAGTGATGCCG -3’ |
|  | Reverse | 5’ -CTGCTGTGGTCATCAGGTAGG -3’ |
| Mouse *Irg1* | Forward | 5’ -GGTATCATTCGGAGGAGCAAGAG -3’ |
|  | Reverse | 5’ -CCCAAACAGTGCTGGAGGTGTTG -3’ |
| Mouse *Hmox1* | Forward | 5’ -CGAATGAACACTCTGGAGATGACAC -3’ |
|  | Reverse | 5’ -GTGTTCCTCTGTCAGCATCACC -3’ |
| Mouse *Gapdh* | Forward | 5’ -CTTAAGAGGGATGCTGCCCTTACC -3’ |
|  | Reverse | 5’ -CCAATACGGCCAAATCCGTTCACAC -3’ |
