# Supplementary figures and images for: Atractylodes lancea (Thunb.) DC. [Asteraceae] rhizome-derived exosome-like nanoparticles suppress lipopolysaccharide-induced inflammation in murine microglial cells
Source: Front Pharmacol. 2024 Apr 26;15:1302055. doi: 10.3389/fphar.2024.1302055 (PMC11082290; doi:10.3389/fphar.2024.1302055)

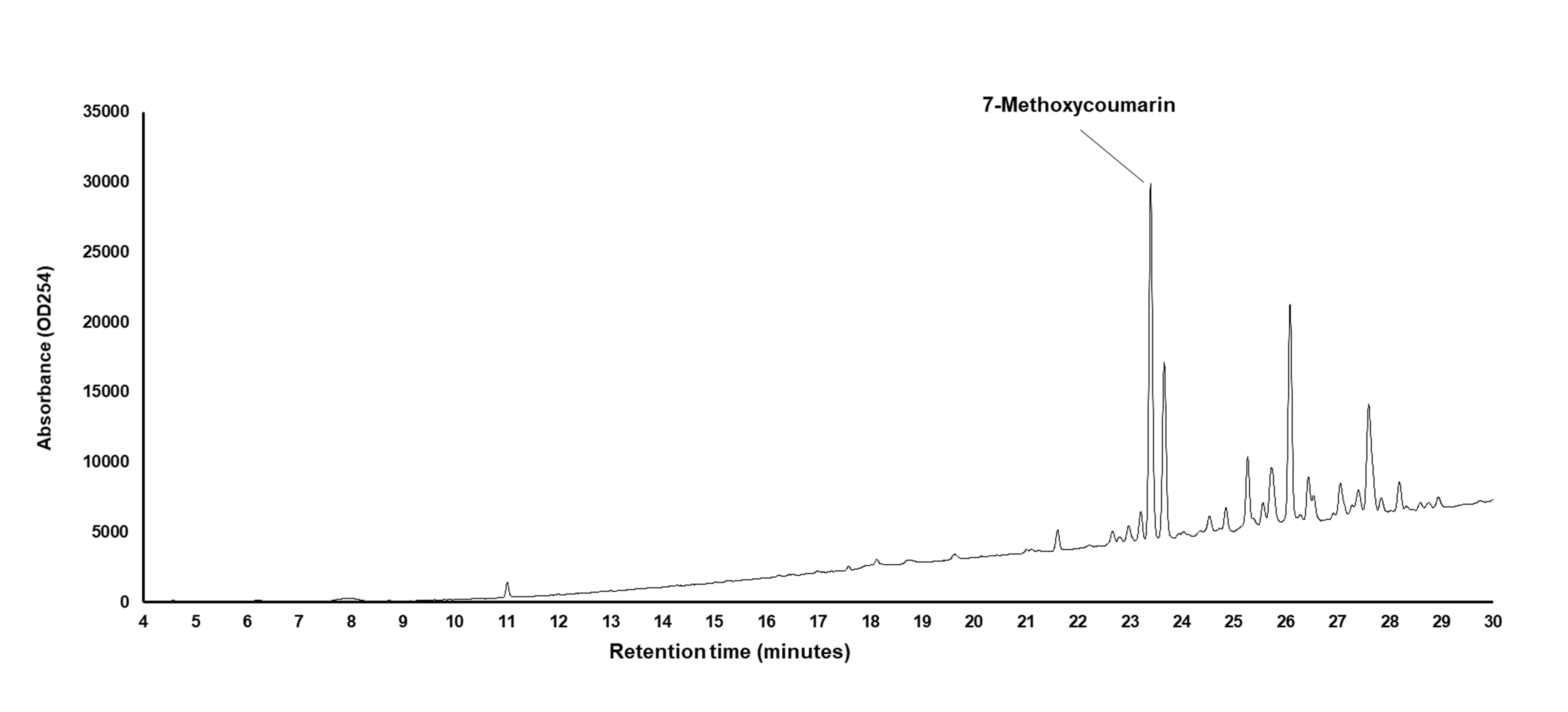

Supplement: Supplementary file 2 [file Image1.TIF]
